# Systematic, active surveillance for Middle East respiratory syndrome coronavirus in camels in Egypt

**DOI:** 10.1038/emi.2016.130

**Published:** 2017-01-04

**Authors:** Mohamed A Ali, Mahmoud M Shehata, Mokhtar R Gomaa, Ahmed Kandeil, Rabeh El-Shesheny, Ahmed S Kayed, Ahmed N El-Taweel, Mohamed Atea, Nagla Hassan, Ola Bagato, Yassmin Moatasim, Sara H Mahmoud, Omnia Kutkat, Asmaa M Maatouq, Ahmed Osman, Pamela P McKenzie, Richard J Webby, Ghazi Kayali

**Affiliations:** 1 Center of Scientific Excellence for Influenza Viruses, National Research Centre, Giza 12311, Egypt; 2 General Organization of Veterinary Services, Ministry of Agriculture and Land Reclamation, Giza 11772, Egypt; 3 Department of Biochemistry, Faculty of Science, Ain Shams University, Cairo 38105, Egypt; 4 Department of Infectious Diseases, St Jude Children's Research Hospital, Memphis, TN 77030, USA; 5 Department of Epidemiology, Human Genetics, and Environmental Sciences, University of Texas, Houston, TX 77030, USA; 6 Human Link, Baabda 1107-2090, Lebanon

**Keywords:** camel, Egypt, Middle East respiratory syndrome coronavirus, surveillance

## Abstract

Middle East respiratory syndrome coronavirus (MERS-CoV) causes severe human infections and dromedary camels are considered an intermediary host. The dynamics of natural infection in camels are not well understood. Through systematic surveillance in Egypt, nasal, rectal, milk, urine and serum samples were collected from camels between June 2014 and February 2016. Locations included quarantines, markets, abattoirs, free-roaming herds and farmed breeding herds. The overall seroprevalence was 71% and RNA detection rate was 15%. Imported camels had higher seroprevalence (90% vs 61%) and higher RT-PCR detection rates (21% vs 12%) than locally raised camels. Juveniles had lower seroprevalence than adults (37% vs 82%) but similar RT-PCR detection rates (16% vs 15%). An outbreak in a breeding herd, showed that antibodies rapidly wane, that camels become re-infected, and that outbreaks in a herd are sustained for an extended time. Maternal antibodies titers were very low in calves regardless of the antibody titers of the mothers. Our results support the hypothesis that camels are a reservoir for MERS-CoV and that camel trade is an important route of introducing the virus into importing countries. Findings related to waning antibodies and re-infection have implications for camel vaccine development, disease management and zoonotic threat.

## INTRODUCTION

The Middle East respiratory syndrome (MERS) is caused by a betacoronavirus (βCoV) and was first reported infecting a human being in 2012.^1^ As of 22 July 2016, the World Health Organization (WHO) was notified of 1782 cases of whom 634 died.^2^ The majority of these cases originate in the Arabian Peninsula especially Saudi Arabia. Travel-related cases have occurred in more than 20 countries and recently a major outbreak was recorded in South Korea.^3^

Current evidence suggests that MERS-CoV is a zoonotic virus. MERS-CoV is genetically related to bat βCoV.^1, 4, 5, 6^ Bats were also found susceptible to infection by MERS-CoV.^7^ More compelling evidence of zoonotic transmission suggests that dromedary camels are a reservoir for MERS-CoV. Several studies detected MERS-CoV in camels in several countries.^8, 9, 10, 11^ Camel to human transmission of MERS-CoV was also documented.^12, 13^ Camel and human MERS-CoV had similar replication, tropism and ability to evade interferon responses, in *ex vivo* cultures of the human respiratory tract. Yet, it is important to note that the vast majority of the human cases occur after human-to-human transmission especially in health care settings.^3^

The role of camels as a MERS-CoV reservoir was initially supported by the prevalence of MERS antibodies in camels from Oman, Egypt and Saudi Arabia.^14, 15, 16^ It was later shown that dromedary camels from African countries (Ethiopia, Kenya, Nigeria, Sudan and Tunisia) and Arabian Peninsula (Jordan, Oman, Qatar and United Arab Emirates (UAE)) had high seropositive rates of MERS-CoV antibodies.^17^ Archived camel sera (1983–1997) showed the presence of neutralizing antibodies suggesting long-term MERS-CoV circulation among camels.^18^

However, most of the evidence relating camels to MERS-CoV comes from cross-sectional studies that do not provide information on the dynamics of MERS-CoV infection in camels. Furthermore, the majority of traded camels originate from African countries and those have not been well studied. Hence, we designed a systematic active surveillance system to study MERS-CoV in camels, both imported from Sudan and local, in Egypt. The aim here was to determine whether the prevalence of MERS-CoV differed by settings at which camels are raised.

## MATERIALS AND METHODS

### Samples and locations

**Figure 1:**
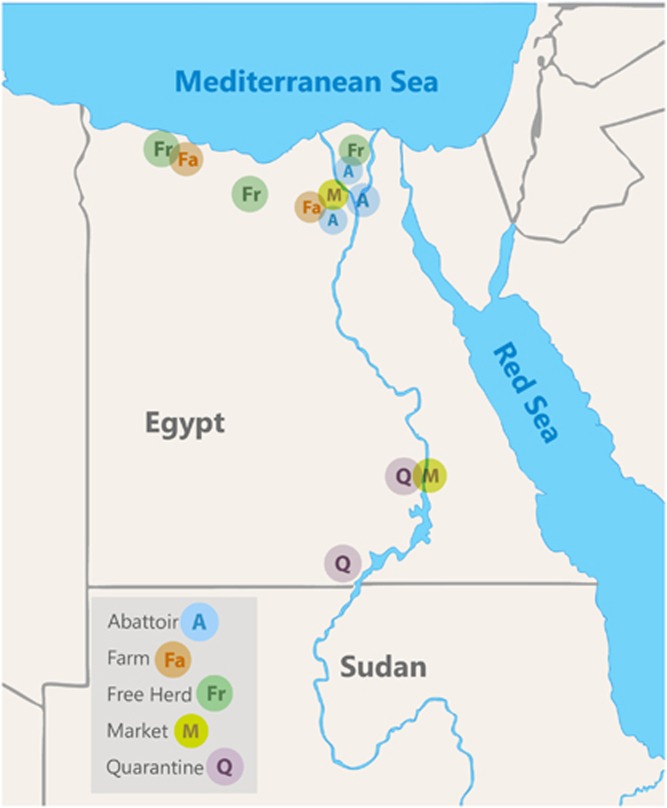
Map of sampling locations. Quarantines were located near the border with Sudan, abattoirs were in Cairo and the Nile Delta region, free herds and farms were in Northern Egypt.

Between June 2014 and February 2016, 2825 nasal swabs, 114 rectal swabs, 187 milk samples, 26 urine samples and 2541 sera were collected from dromedary camels. Sampling locations are shown in Figure 1 and included a government-operated quarantine at the border with Sudan, two camel markets and camel abattoirs where imported camels mainly from Sudan, Somalia and Ethiopia are found. Local free-roaming herds, and local farmed breeding herds including a herd of about 90 camels that were raised and bred in confinement on the Mediterranean coast were also sampled repeatedly every month. Camels were classified as juvenile when under two years of age around when they are weaned off mother's milk.

Nasal and rectal swabs, urine and milk samples were tested for the presence of MERS-CoV while milk and serum samples were tested for the presence of MERS antibodies. All procedures involving animals were done in accordance with the Guide for the Care and Use of Laboratory Animals and were approved by the Institutional Animal Care and Use Committee at St Jude Children's Research Hospital, Memphis, TN, USA.

### Serological testing

The microneutralization assay was used to determine antibodies against MERS-CoV. The assay was conducted as previously described and positive and negative camel antisera were included in all runs.^14^ Results were only accepted if results from the positive and negative antisera were as expected. Test samples (milk and sera) were initially screened at a dilution of 1:10 in duplicate. Any sample that had a positive result when screened was then repeated in duplicate to obtain the end-point titer that was expressed as the reciprocal of the dilution that provided complete neutralization. Titers ≥1:20 were considered positive.

### Molecular testing

The WHO testing algorithm for MERS-CoV was implemented.^19^ Viral RNA was extracted from nasal, rectal, milk and urine samples using QIAmp viral RNA minikit (Qiagen, Dusseldorf, Germany). RNA was then tested for the presence of MERS-CoV RNA using the *upE* real-time RT-PCR assay as described previously.^20^ Samples testing positive by the *upE* assay regardless of CT-value were then confirmed by at least one other RT-PCR assay including the *ORF1a*, *RdRpseq* and *Nseq* assays as described previously.^21^

Sanger sequencing was performed as part of the confirmatory *RdRpseq* and *Nseq* assays. Partial Spike protein gene sequences for genotyping were also obtained for 21 viruses (1 from a local camel and 20 from imported camels) from nasal swabs as per a previously published protocol.^22^ These 21 sequences were submitted to GenBank under accession numbers KU942355-KU942375. Phylogenetic tree of the partial Spike protein gene (around 600 bp) was constructed using MEGA 6 with bootstrap method and Kimura 2-parameter model.^23^

### Statistical analysis and figure development

Chi-square and Fisher's exact tests were used to perform the statistical comparisons. Statistical significance was set at a *P*-value <0.05. Analysis was performed using SPSS v18 (IBM, Armonk, NY, USA).

## RESULTS

### Overall

The distribution of sera and nasal swabs by sampling site, age, sex and animal origin is presented in Table 1. The majority of sera were from farmed dromedary camels (54%), adults (77%) and local (65%). Sera were obtained from 1254 males (49%) and 1090 females (43%), but sex data for 197 (8%) samples were missing. A majority of nasal swabs were obtained from mostly farm camels (49%), adults (79%) and local camels (59%). Nasal swabs were sampled from 1439 males (51%) and 1089 females (39%), but sex data for 297 (11%) samples were missing.

Overall seroprevalence and MERS-CoV RNA detection rates are shown in Table 1. Of the 2541 serum samples tested, 1808 (71%) tested positive. Camels sampled at quarantines and markets had the highest seroprevalence (>92%), while camels from farms had the lowest seroprevalence (<60%) (*P*-value <0.0001). Juvenile camels had a significantly lower seroprevalence than adult camels (37% and 82%, respectively, *P*-value <0.0001). The sex of the camel was a determinant of seroprevalence as males tended to have higher titers than females (*P*-value=0.003). Imported camels, mainly from Sudan, Somalia and Ethiopia, had a significantly higher seropositivity rate as compared with local camels (90% and 61%, respectively, *P*-value <0.0001). Moreover, imported camels had higher titers as 52% of them had titers ≥1:160 as compared with 30% of local camels (*P*-value <0.0001; data not shown).

MERS-CoV RNA detection rate from nasal swabs was 15% overall (435 positive samples out of 2825 tested; Table 1). The virus' RNA was detected in all sampling sites but was highest in quarantines (36%) and lowest among free herd camels (1% *P*-value <0.0001). Rates of viral RNA detection from juveniles and adults were not different. However, more male camels tested positive than females (*P*-value <0.0001). MERS-CoV was detected two times more in imported camels than local ones (21% and 12%, respectively, *P*-value <0.0001).

**Figure 2:**
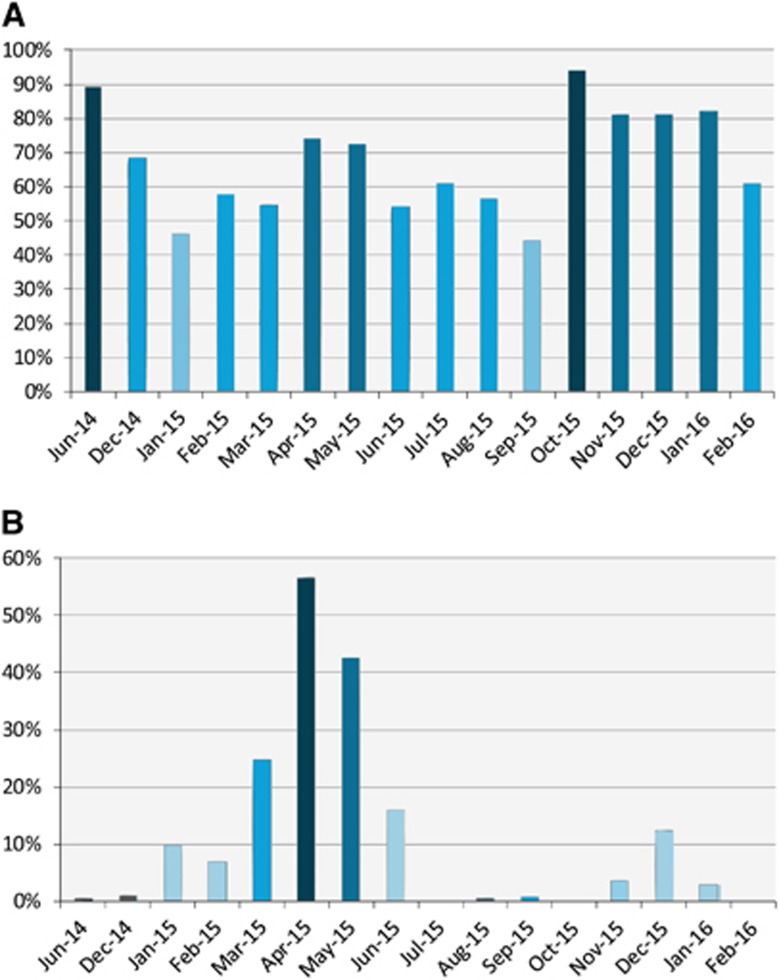
Serological and viral RNA detection distribution of MERS-CoV in camels in Egypt over time. (**A**) Distribution of antibody titers by month. The *y* axis shows the percentage of seropositive samples (neutralizing antibody titer ≥1:20). (**B**) Detection of viral nucleic acids by month. The *y* axis shows the percentage of nasal swabs with confirmed MERS-CoV RNA by RT-PCR. All samples were included in the analysis. Supporting data are provided in Supplementary Table S2. Middle East respiratory syndrome coronavirus, MERS-CoV; polymerase chain reaction with reverse transcription, RT-PCR.

Seroprevalence and detection of MERS-CoV RNA in nasal swabs varied by month (Figure 2). Viral RNA detection increased in the winter months (December, January and February). Following January 2015, viral RNA detection continued to increase through April 2015 when it peaked at 57% then started to drop.

**Figure 3:**
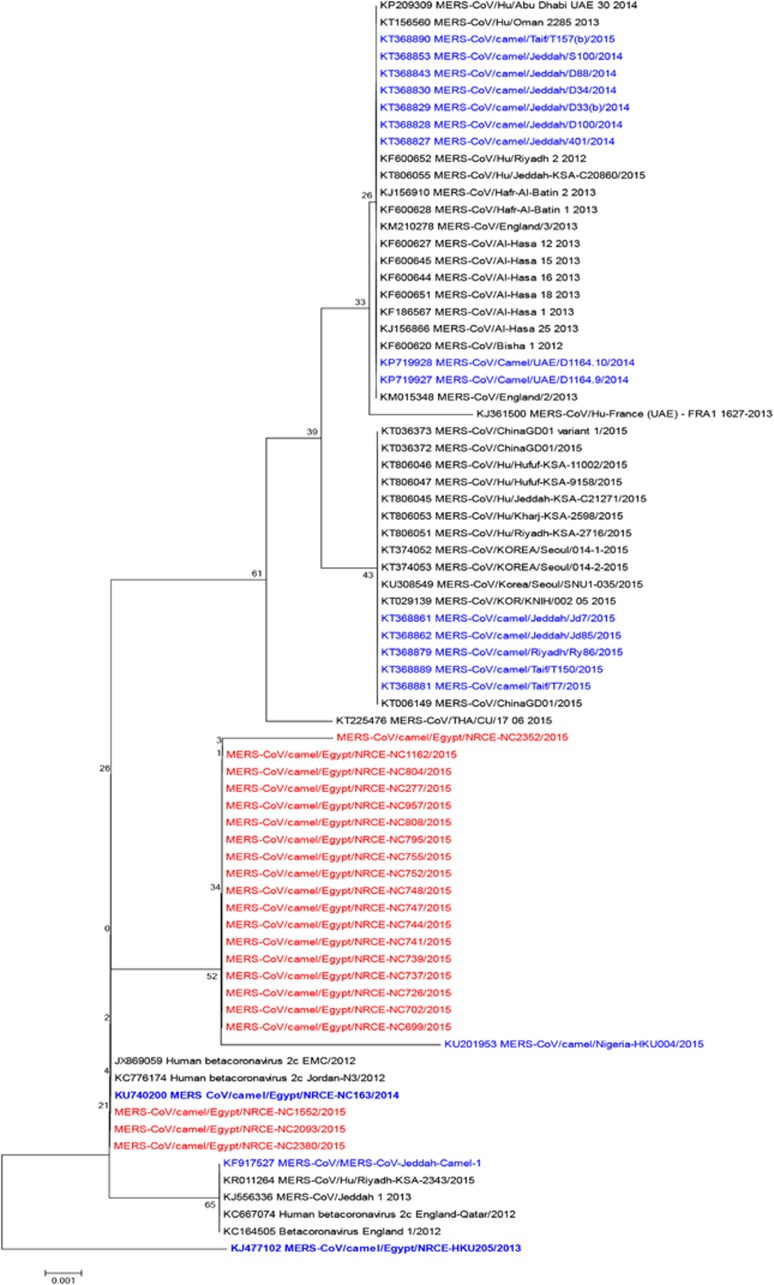
Phylogenetic tree of the partial Spike protein gene (around 600 bp) of MERS-CoV. Tree was generated using MEGA6 with bootstrap method and Kimura 2-parameter model. MERS-CoV/Camel/Egypt/NRCE-HKU205/2013 (KJ477102) was used as a root for the tree. Sequences obtained in this study are shown in red, other camel sequences are shown in blue. Middle East respiratory syndrome coronavirus, MERS-CoV.

The phylogenetic tree for the partial Spike gene, that is used for virus genotyping, is shown in Figure 3. Sequenced viruses clustered within clade A MERS-CoV regardless of camel origin or time of sampling. Three sequences were closely related to the initial human MERS-CoV isolates, while the rest clustered together and were similar to a 2015 camel isolate from Nigeria.^24^

### Comparison between imported and local camels

**Figure 4:**
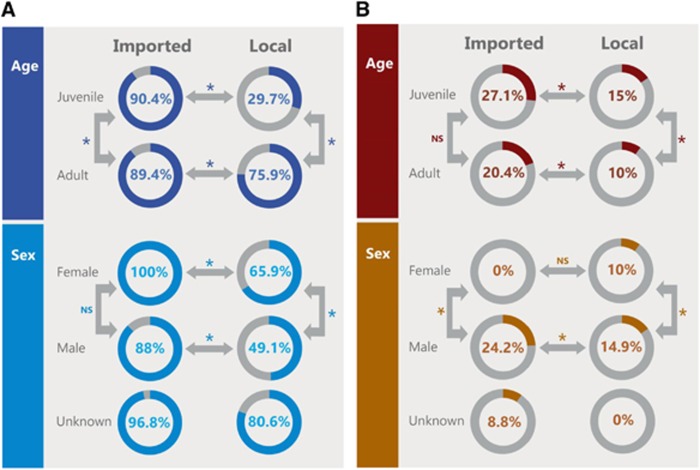
(**A**) Comparison of seroprevalence between local and imported camels by age and sex. Numbers in the circles indicate the percentage of seropositive samples (neutralizing antibody titer ≥1:20). Arrows indicate categories compared with each other. Statistically significant differences (*P*-value <0.05) are denoted by *. NS denotes not statistically significant. (**B**) Comparison of detection of viral RNA between local and imported camels by age and sex. Numbers in the circles indicate the percentage of nasal swabs with confirmed MERS-CoV RNA by RT-PCR. Arrows indicate categories compared with each other. Statistically significant differences (*P*-value <0.05) are denoted by *. NS denotes not statistically significant. Middle East respiratory syndrome coronavirus, MERS-CoV; polymerase chain reaction with reverse transcription, RT-PCR.

Local juvenile camels had the lowest seroprevalence (30%), significantly different than local adults (76%) and imported juveniles (90% Figure 4A). Imported adults had a significantly higher antibody prevalence than local camels. Male local camels had the lowest seroprevalence (49%) that was significantly different than that among the local females (66%) and the imported males (88%). All imported female camels were seropositive and this was significantly different than the local females. There was no statistical difference in seroprevalence between female and male imported camels.

**Figure 5:**
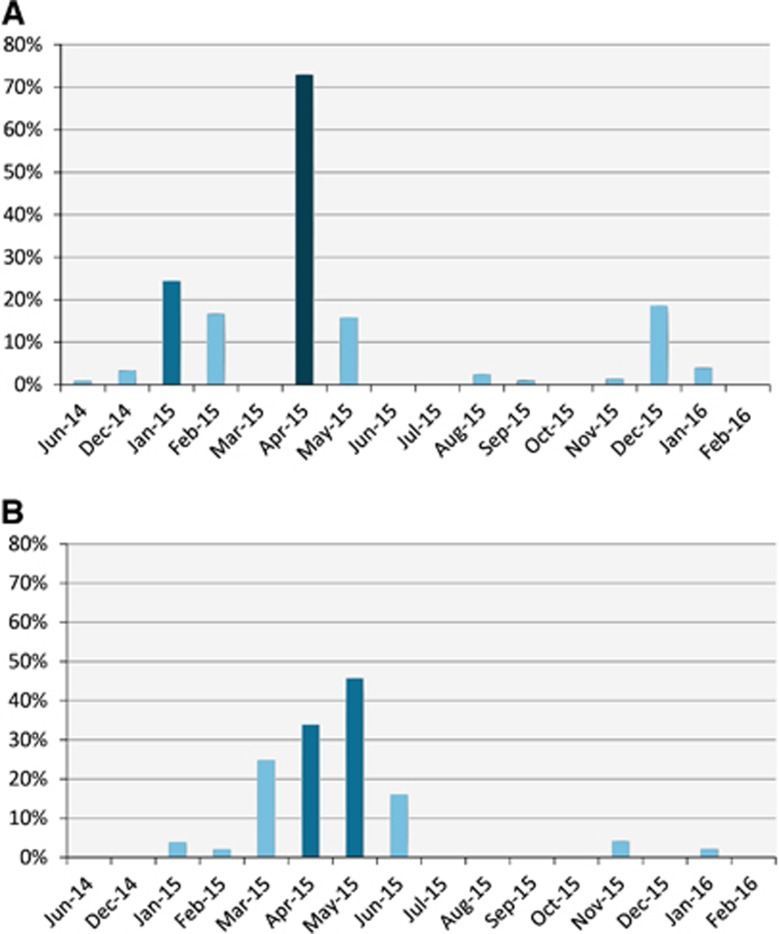
Detection of viral RNA in imported (**A**) and local (**B**) camels over the surveillance period. *Y* axes show the percentage of nasal swabs with confirmed MERS-CoV RNA by RT-PCR. Supporting data are provided in Supplementary Table S3. Middle East respiratory syndrome coronavirus, MERS-CoV; polymerase chain reaction with reverse transcription, RT-PCR.

Figure 4B shows the distribution of RNA detection rate by age and sex. Juvenile imported camels had the highest rate (27%), significantly higher than the local juveniles (15%) but not significantly higher than the imported adults (20%). Local adult camels had the lowest rate (10%), statistically lower than local juvenile and imported adults. Females had the lowest rate regardless of source of animal (0% and 10% for imported and local, respectively) and were significantly lower than males. Imported males had the highest rates (24%) significantly higher than their local counterparts (15%). RNA detection rates appeared to be higher in winter and spring months irrespective of camel origin (Figure 5A for imported camels and Figure 5B for local ones).

MERS-CoV RNA was detected in 18 of 114 rectal swabs (15.4%), in 12 of 187 milk samples (6.4%) and in 0 of 26 urine samples. Antibodies were detected in 38 of 187 milk samples (20.3%) though in low titers (geometric mean titer=21.5).

### Local farm camels

**Figure 6:**
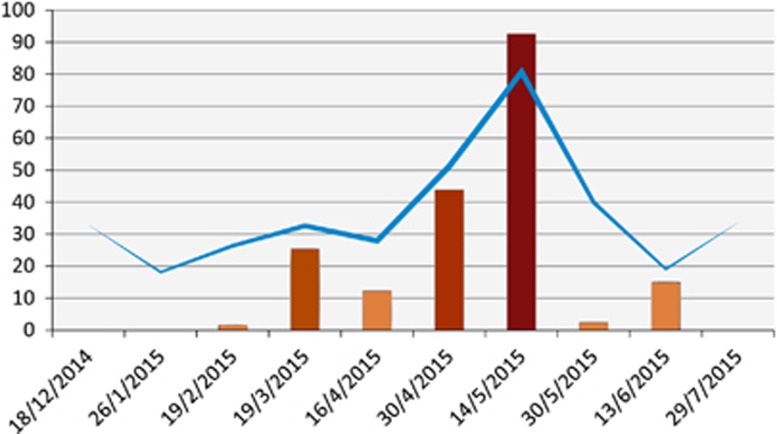
Epicurve of MERS-CoV outbreak in a local herd and the associated antibody geometric mean titers. Bars indicate the percentage of nasal swabs with confirmed MERS-CoV RNA by RT-PCR. Line indicates the geometric mean of antibody titers. Supporting data are provided in Supplementary Table S4. Middle East respiratory syndrome coronavirus, MERS-CoV; polymerase chain reaction with reverse transcription, RT-PCR.

A herd of about 90 locally bred camels residing on a farm on the Mediterranean coast was closely surveyed (Figure 6). MERS-CoV RNA was first detected on this farm on 19 February 2015 in one adult camel. One month later, viral RNA was detected in 16 camels (13 adults and three juveniles) and thus frequency of sampling was increased to every two weeks. In mid-April, nine camels were positive (three adults and six juveniles). Two weeks later, 44% of the herd was positive and then 93% were positive two weeks after (May 14). The rate dropped to 2.5% on May 30, then 15% on June 13. After that, the herd became negative and continued to be so until the last sample collection visit in February 2016. Hence, the outbreak lasted for nearly four months.

At the onset of the outbreak, the geometric mean titer of antibodies within the herd was 1:18. As the outbreak advanced, antibody titers increased and peaked at a geometric mean of 1:80 on May 14. Titers waned off rapidly as the outbreak slowed down returning back to the same level as before the outbreak. Camels that were seronegative at the onset of the outbreak sero-converted during it. Although camels that were seropositive at onset had 1–3-fold increase in titer. After peaking, antibody titers dropped by 1–4 folds at the sampling point immediately after (Supplementary Table S1).

Maternal antibodies were measured in 11 calf–mother pairs (Table 2A). All calves had antibody titers ≤1:20 regardless of the antibody titers of the mother. We recorded re-infection of camels with MERS-CoV. Four camels tested positive before the detection of the outbreak (January 30) and became infected again during the outbreak. All but one camel showed sero-conversion (Table 2B).

## DISCUSSION

Surveillance is key to understand the epidemiology and epizootology of emerging zoonotic viruses. Here we conducted systematic active surveillance in Egyptian camels aimed at determining the incidence and seroprevalence of MERS-CoV. Our overall seroprevalence data are in line with previous findings from Egypt and elsewhere.^17^ MERS-CoV detection rates by RT-PCR is also comparable to studies in Saudi Arabia and the UAE.^10, 11^

Egypt imports around 1.2 million heads of camels annually, of which 68% are from Sudan and the rest from East African countries, primarily Ethiopia.^25^ When comparing local with imported camels, our data showed that imported camels had higher seroprevalence and higher RT-PCR detection rates. Seroprevalence in imported camels was comparable to a previous study of imported camels in Egypt as well as camels tested in other countries.^26^ Conducting surveillance at quarantines and adjacent markets in Southern Egypt meant that we were sampling the animals as soon as they arrived from Sudan in Egypt, hence antibody titers and infections occurred prior to the camels arriving in Egypt. This suggests that MERS-CoV is being introduced through camel trade. Our surveillance included four sampling trips to those locations and the majority of the sampled camels were seropositive. RT-PCR detection rate ranged between 0% and 2.7% for three trips, however, 86% of the camels sampled on the fourth trip were positive.

Our data also supported the observation that juvenile camels are more susceptible to infection due to low seroprevalence.^8, 10, 11^ We were able to demonstrate that several of those camels have no maternal antibodies by the end of the first month of age even when born to seropositive mothers. This conclusion was demonstrated by a previous study from the UAE.^27^ Recent data demonstrated that MERS-CoV neutralizing antibody was detected in camel milk.^15^ Our data also showed that antibody titers in milk are uncommon and are at low titers when detected. Furthermore, the outbreak we detected in the local farmed herd occurred immediately after and during the time when calves were born (December–April). MERS-CoV detection by RT-PCR was higher in juvenile camels regardless of origin.

More female camels were seropositive than males when the analysis was performed by camel origin, that is, imported vs local. Females had consistently higher seroprevalence even when data were further analyzed by age strata. This translated into lower detection of MERS-CoV in females as compared with males. Our data does not allow us to provide a biological explanation for this observation, however, it could be potentially explained by the close link of cows and calves. As calves are more susceptible to infection, the cows become exposed to virus repeatedly, increasing their antibody titer.

Phylogenetic analysis of a portion of the Spike gene showed that MERS-CoV in camels in Egypt belongs to clade A that is related to early human MERS-CoV viruses and includes a camel MERS-CoV sequence from Nigeria. This is distinct from clade B viruses that are causing the recent human infections and that were found to be infecting camels in Saudi Arabia and the UAE. Given the size of the MERS-CoV genome, we cannot draw much conclusions on the genetic composition of Egyptian viruses based on only partial Spike sequences. However, we are in the process of obtaining full genome sequences of the viruses we detected in our surveillance.

Monitoring the outbreak of MERS-CoV in the local farm herd enabled us to learn two novel findings: (i) that outbreaks in camel herds last a long time, and (ii) that antibody titers rapidly drop within a period often as short as two weeks. Furthermore, we were able to demonstrate that re-infection of camels does happen based on increases in antibody titers. This suggests that the virus may be continuously circulating in the large herds in exporting countries. These data have implications on vaccinating camels against MERS-CoV. Any vaccination program should account for the facts that antibodies tend to wane and that camels with high antibody titers can be infected.^9, 10^ This is in addition to the fact that the infection appears to be more common in exporting countries from which data on camel populations are sparse. Another study demonstrated that MERS neutralizing antibodies in dairy camels in a high biosecurity level farm remained consistent for up to one year.^27^ This is in contrast to our serological data that showed variation of antibodies over time. This may be explained but differences in biosecurity and/or differences in the immunity of the camels given different feeding and rearing practices.

Data shown in this report indicate that MERS-CoV is common in camels in Egypt and those imported from other African countries. Yet to date, camel-to-human transmission of the virus has not been reported in this country. Only one human case of an Egyptian who became symptomatic upon return from Saudi Arabia was reported. Similarly, travel-associated cases were reported in Algeria and Tunisia but none were reported in any other African country. Comparatively, camel-to-human transmission is common in the Arabian Peninsula where most of the human cases are reported. Whether this difference is related to viral genetic factors, human behavior, public health surveillance and healthcare delivery, or other factors is a major question that is yet to be answered.

Our results support the observation that camels are a reservoir for MERS-CoV. The possibility of re-infection and the waning antibodies suggests that MERS-CoV may be circulating in enzootic levels in camels from Sudan, or at least some regions in Sudan, as well as other camel producing countries in Africa. Hence, surveillance in those countries is of utmost importance.

## Figures and Tables

**Table 1:** MERS-CoV microneutralization and nasal swab RT-PCR test results by sampling site, age, sex and animal origin

**Sampling site**	**Microneutralization assay**		**Nasal swabs RT-PCR**	
	**Number tested (%)**	**Number (%) positive**	**Number tested (%)**	**Number (%) positive**
Live animal market	172 (6.8%)	159 (92.4%)	159 (5.6%)	4 (2.5%)
Free herd	282 (11.1%)	202 (71.6%)	282 (10.0%)	3 (1.1%)
Farm	1373 (54.0%)	813 (59.2%)	1376 (48.7%)	189 (13.7%)
Quarantine	361 (14.2%)	342 (94.7%)	424 (15.0%)	153 (36.1%)
Slaughterhouse	353 (13.9%)	292 (82.7%)	584 (20.7%)	86 (14.7%)
*P-*value		<0.0001		<0.0001
*Age*				
Juvenile	595 (23.4%)	221 (37.1%)	591 (20.9%)	97 (16.4%)
Adult	1946 (76.6%)	1587 (81.6%)	2234 (79.1%)	338 (15.1%)
*P*-value		<0.0001		NS
*Sex*				
Male	1254 (49.4%)	905 (72.2%)	1439 (50.9%)	300 (20.8%)
Female	1090 (42.9%)	724 (66.4%)	1089 (38.6%)	115 (10.6%)
Unknown	197 (7.7%)	179 (90.9%)	297 (10.5%)	20 (6.7%)
*P-*value		<0.0001^a^		<0.0001^b^
*Animal origin*				
Imported	886 (34.9%)	793 (89.5%)	1167 (41.3%)	243 (20.8%)
Local	1655 (65.1%)	1015 (61.3%)	1658 (58.7%)	192 (11.6%)
*P-*value		<0.0001^a^		<0.0001^a^
Total	2541	1808 (71.2%)	2825	435 (15.40%)

Abbreviations: Middle East respiratory syndrome coronavirus, MERS-CoV; not significant, NS.

A microneutralization positive sample was that with a neutralizing antibody titer ≥1:20. A positive RT-PCR sample was positive by the *upE* RT-PCR assay and another confirmatory RT-PCR assay.

a *P*-value is 0.003 when the unknown category is excluded.

b *P*-value is <0.0001 when the unknown category is excluded.

**Table 2A:** MERS-CoV neutralizing antibody titers in matched mother–calf pairs

**Calf date of birth**	**Calf sampling date**	**Calf age at sample (days)**	**Calf titer**	**Mother sampling date**	**Mother titer**
30/11/14	18/12/14	19	5	18/12/14	160
27/12/14	26/1/15	30	5	18/12/14	40
02/1/15	26/1/15	24	5	18/12/14	40
10/1/15	26/1/15	16	5	18/12/14	320
19/1/15	16/4/15	87	5	18/12/14	80
20/1/15	16/4/15	86	20	18/12/14	320
23/1/15	16/4/15	81	5	18/12/14	640
26/1/15	16/4/15	68	10	26/1/14	5
27/1/15	16/4/15	67	10	26/1/14	80
03/2/15	19/2/15	16	5	26/1/14	20
14/2/15	19/2/15	5	20	26/1/14	320

**Table 2B:** Neutralizing antibody titers against MERS-CoV in re-infected camels

**Time between first and second seropositive result (days)**	**Antibody titer at first seropositive result**	**Antibody titer after four weeks of first seropositive result**	**Antibody titer at second seropositive result**	**Antibody titer after four weeks of second seropositive result**
55	1:80	1:160	1:40	1:80
48	Negative	Negative	Negative	Negative
69	Negative	Negative	1:40	1:160
48	Negative	Negative	1:160	Negative
