# Supplementary material for: Systematic, active surveillance for Middle East respiratory syndrome coronavirus in camels in Egypt
Source: Emerg Microbes Infect. 2017 Jan 4;6(1):e1–. doi: 10.1038/emi.2016.130 (PMC5285495; doi:10.1038/emi.2016.130)
Supplement: Supplementary Table S1 [file emi2016130x1.pdf]

Supplementary Table S1 Serolgical response of selected camels during an outbreak of MERS-CoV in a local herd.

18/12/2014	26/1/2015	19/2/2015	19/3/2015	16/4/2015	30/4/2015	14/5/2015	30/5/2015	13/6/2015	29/7/2015	Sex	Age
neg	neg	20	160	160	160	80	20	80	40	F	Adult
40	40	80	20	20	320	40	160	neg	10	F	Adult
20	neg	20	640	160	40	1280	40	neg	40	F	Adult
40	40	80	40	20	80	80	80	20	40	F	Adult
20	neg	40	20	80	40	160	40	40	40	F	Adult
20	neg	40	40	10	40	640	20	20	10	F	Adult
40	neg	40	40	40	80	1280	40	10	20	F	Adult
160	160	20	20	40	320	1280	320	10	160	F	Adult
neg	neg	20	160	20	20	40	20	40	80	F	Adult
40	40	80	320	10	80	160	40	neg	20	F	Adult
80	40	80	160	80	160	320	160	20	20	F	Adult
neg	neg	neg	10	80	10	160	10	10	160	F	Adult
20	20	20	20	20	40	20	80	80	160	F	Adult
80	80	20	40	40	320	320	160	20	80	F	Adult
80	80	160	160	neg	40	320	320	80	80	F	Adult
160	neg	80	neg	40	320	160	160	10	160	F	Adult
40	neg	10	320	80	160	320	160	40	160	F	Adult
160	80	160	160	10	320	160	160	40	640	F	Adult
160	80	80	80	160	160	20	80	160	40	F	Adult
40	neg	40	20	neg	80	320	40	10	160	F	Adult
40	160	160	160	160	640	10	320	neg	20	F	Adult
80	20	80	20	20	160	20	160	40	1280	F	Adult
320	40	neg	20	160	160	320	30	neg	40	F	Adult
40	neg	80	640	10	160	80	80	40	80	F	Adult
20	neg	20	40	20	40	neg	20	neg	10	F	Adult
320	320	160	40	320	1280	1280	640	40	640	F	Adult
160	160	160	80	160	320	80	80	80	10	F	Adult
neg	neg	neg	neg	neg	20	neg	neg	20	neg	F	Adult
neg	neg	neg	neg	40	neg	neg	neg	40	neg	M	Juvenile
neg	neg	neg	40	neg	neg	160	neg	neg	neg	F	Juvenile
neg	neg	neg	160	neg	neg	neg	neg	neg	80	F	Juvenile
neg	neg	neg	80	40	neg	neg	neg	40	40	M	Juvenile
