# Supplementary material for: Systematic, active surveillance for Middle East respiratory syndrome coronavirus in camels in Egypt
Source: Emerg Microbes Infect. 2017 Jan 4;6(1):e1–. doi: 10.1038/emi.2016.130 (PMC5285495; doi:10.1038/emi.2016.130)
Supplement: Supplementary Table S2 [file emi2016130x2.pdf]

Supplementary Table S2. Supporting data for Figure 2.

A

Time	Total no. tested	No. positive	percentage
Jun-14	130	116	89.20%
Dec-14	92	63	68.50%
Jan-15	119	55	46.20%
Feb-15	109	63	57.80%
Mar-15	117	64	54.70%
Apr-15	269	199	74.00%
May-15	163	118	72.40%
Jun-15	81	44	54.30%
Jul-15	82	50	61.00%
Aug-15	175	99	56.60%
Sep-15	120	53	44.20%
Oct-15	112	105	93.80%
Nov-15	279	227	81.40%
Dec-15	296	241	81.40%
Jan-16	328	269	82.00%
Feb-16	69	42	60.90%
**Total**	**2541**	**1808**	**%71.20**

B

Time	Total no. tested	No. positive	percentage
Jun-14	157	1	%0.60
Dec-14	104	1	%1
Jan-15	144	14	%9.70
Feb-15	143	10	%7
Mar-15	117	29	%24.80
Apr-15	401	227	%56.60
May-15	183	78	%42.60
Jun-15	81	13	%16
Jul-15	82	0	%0
Aug-15	161	1	%0.60
Sep-16	151	1	%0.70
Oct-15	112	0	%0
Nov-15	260	9	%3.50
Dec-15	329	41	%12.50
Jan-16	329	10	%3
Feb-16	71	0	%0
**Total**	**2825**	**435**	**%15.40**
