# Supplementary material for: Systematic, active surveillance for Middle East respiratory syndrome coronavirus in camels in Egypt
Source: Emerg Microbes Infect. 2017 Jan 4;6(1):e1–. doi: 10.1038/emi.2016.130 (PMC5285495; doi:10.1038/emi.2016.130)
Supplement: Supplementary Table S3 [file emi2016130x3.pdf]

**Supplementary Table S3 Supporting data for Figure 5.**

**A**

| Time | Total no. tested | No. positive | percentage |
|---|---|---|---|
| Jun-14 | 107 | 1 | %0.90 |
| Dec-14 | 30 | 1 | %3.30 |
| Jan-15 | 41 | 10 | %24.40 |
| Feb-15 | 48 | 8 | %16.70 |
| Mar-15 | | | |
| Apr-15 | 233 | 170 | %73.00 |
| May-15 | 19 | 3 | %15.80 |
| Jun-15 | | | |
| Jul-15 | | | |
| Aug-15 | 40 | 1 | %2.50 |
| Sep-16 | 97 | 1 | %1.00 |
| Oct-15 | 112 | 0 | %0.00 |
| Nov-15 | 69 | 1 | %1.40 |
| Dec-15 | 221 | 41 | %18.60 |
| Jan-16 | 150 | 6 | %4.00 |
| Feb-16 | | | |
| Total | 1167 | 243 | 20.82% |

**B**

| Time | Total no. tested | No. positive | percentage |
|---|---|---|---|
| Jun-14 | 50 | 0 | 0.00% |
| Dec-14 | 74 | 0 | 0.00% |
| Jan-15 | 103 | 4 | 3.90% |
| Feb-15 | 95 | 2 | 2.10% |
| Mar-15 | 117 | 29 | 24.80% |
| Apr-15 | 168 | 57 | 33.90% |
| May-15 | 164 | 75 | 45.70% |
| Jun-15 | 81 | 13 | 16.00% |
| Jul-15 | 82 | 0 | 0.00% |
| Aug-15 | 121 | 0 | 0.00% |
| Sep-16 | 54 | 0 | 0.00% |
| Oct-15 | | | |
| Nov-15 | 191 | 8 | 4.20% |
| Dec-15 | 108 | 0 | 0.00% |
| Jan-16 | 179 | 4 | 2.20% |
| Feb-16 | 71 | 0 | 0.00% |
| Total | 1658 | 192 | 11.58% |
