# Supplementary material for: Systematic, active surveillance for Middle East respiratory syndrome coronavirus in camels in Egypt
Source: Emerg Microbes Infect. 2017 Jan 4;6(1):e1–. doi: 10.1038/emi.2016.130 (PMC5285495; doi:10.1038/emi.2016.130)
Supplement: Supplementary Table S4 [file emi2016130x4.pdf]

Supplementary Table S4 Supporting virological data for Figure 6 (bars).

Time	Total number tested	Number positive	percentage
18/12/2014	60	0	0
26/1/2015	61	0	0
19/2/2015	61	1	1.63934426
19/3/2015	63	16	25.3968254
16/4/2015	73	9	12.32876712
30/4/2015	66	29	43.93939394
14/5/2015	81	75	92.5925926
30/5/2015	79	2	2.53164557
13/6/2015	80	12	15
29/7/2015	80	0	0
